# The impact of SARS-CoV-2 infection in patients with cystic fibrosis undergoing CFTR channel modulators treatment: a literature review

**DOI:** 10.1186/s12931-023-02593-1

**Published:** 2023-11-13

**Authors:** Antonio Vitiello, Michela Sabbatucci, Andrea Silenzi, Annalisa Capuano, Francesco Rossi, Andrea Zovi, Francesco Blasi, Giovanni Rezza

**Affiliations:** 1 Directorate General for Health Prevention, Ministry of Health, Rome, Italy; 2 Department Infectious Diseases, National Institute of Health, Rome, Italy; 3 Section of Pharmacology “L. Donatelli”, Department of Experimental Medicine, Campania Regional Centre for Pharmacovigilance and Pharmacoepidemiology, University of Campania “Luigi Vanvitelli”, Naples, Italy; 4 Directorate General for Hygiene, Food Safety and Nutrition, Ministry of Health, Rome, Italy; 5 Department of Pathophysiology and Transplantation, University of Milan, Milan, Italy; 6 Internal Medicine Department, Respiratory Unit and Cystic Fibrosis Center, Fondazione IRCCS Ca’ Granda Ospedale Maggiore Policlinico, Milan, Italy

**Keywords:** Cystic fibrosis, COVID-19, CFTR modulators, ACE-2, SARS-CoV-2, Angiotensins

## Abstract

Several risk factors for Coronavirus-2019 (COVID-19) disease have been highlighted in clinical evidence. Among the various risk factors are advanced age, metabolic illness such as diabetes, heart disease, and diseases of the respiratory system. Cystic Fibrosis (CF) is a rare disease with autosomal recessive transmission, characterised by a lack of synthesis of the CFTR channel protein, and multi-organ clinical symptoms mainly affecting the respiratory tract with recurrent pulmonary exacerbations. In view of the pathophysiological mechanisms, CF disease should be in theory considered a risk factor for SARS-CoV2 or severe COVID-19. However, recent clinical evidence seems to point in the opposite direction, suggesting that CF could be a protective factor against severe COVID-19. Possibly, the lack of presence or function of the CFTR channel protein could be linked to the expression of the membrane glycoprotein ACE-2, a key enzyme for the endocellular penetration of SARS-CoV-2 and related to the pathophysiology of COVID-19 disease. Furthermore, CFTR channel modulating agents could indirectly influence the expression of ACE-2, playing an important role in restoring the proper functioning of mucociliary clearance and the pulmonary microbiome in the host response to SARS-CoV-2 infection. In this review, the authors attempt to shed light on these important associations of issues that are not yet fully elucidated.

## Introduction

Cystic fibrosis (CF) is a rare multi-organ, autosomal recessive disease caused by mutations of the Cystic Fibrosis Transmembrane Conductance Regulator (CFTR) gene, located in the long arm of chromosome 7 and grouped in five classes. The CFTR gene encodes for chloride and bicarbonate channel expressed on epithelial cells widely in several human tissues. Malfunctioning or downregulation of CFTR channel can affect the respiratory, cardiovascular and gastrointestinal systems. Therefore, patients with CF were expected at increased risk of SARS-CoV-2 infection and severe symptoms of Coronavirus disease 2019 (COVID-19). Strikingly, some studies showed a reduced risk of SARS-CoV-2 infection among CF patients [1] and animal models [2] as well as a role for CFTR in the regulation of SARS-CoV-2 infection and replication of human bronchial epithelial cell lines and primary cells in vitro [3, 4]. In this review we dissected the impact of SARS-CoV-2 infection in patients with CF treated by CFTR channel modulators.

### SARS-CoV-2 infection

SARS-CoV-2 is a virus of the Coronavirus (CoV) family. Coronaviruses (CoVs) are a large family of respiratory viruses with an envelope and a genome consisting of single-stranded positive RNA that can cause mild to moderate illnesses, from the common cold to respiratory syndromes such as MERS (Middle East Respiratory Syndrome), SARS (Severe Acute Respiratory Syndrome) and SARS-CoV-2 [5, 6]. CoVs are common in many animal species (such as camels and bats) but in some cases, although rarely, they can evolve and infect humans. SARS-CoV-2 penetrates the host cell through fusion of the viral envelope with the cell membrane or through membrane fusion within the endosome after endocytosis [7]. One of the main receptors for endocellular penetration used by SARS-CoV-2 is angiotensin-converting enzyme 2 (ACE-2), which is widely expressed in pulmonary pneumocytes, intestinal cells and myocardiocytes. The SARS-CoV-2 virus is responsible for COVID-19 disease that can have an asymptomatic, mild, moderate or even severe course until the patient dies. The typical symptoms of COVID-19, such as dyspnoea, fever, cough and fatigue, can be followed by severe complications, including pneumonia, myocarditis and kidney injury [8]. Since the identification of the first cases in Wuhan, China, to date, the SARS-CoV-2 virus has undergone numerous variations. Some variants are more easily transmissible than others, and some can lead to more serious illnesses. Despite all these mutations, evidence shows that there are a number of risk factors that may increase the likelihood of severe illness or death from COVID-19. These identified risk factors include respiratory diseases, metabolic diseases, heart disease, advanced age, pregnancy, obesity or overweight, and autoimmune diseases [9–11]. Risk factors for severe COVID-19 forms include diseases affecting the respiratory system. Based on these considerations, we could assume that persons with cystic fibrosis (pwCF) might be more susceptible to severe COVID-19 forms. However, some reports have shown that SARS-CoV-2 infection unexpectedly caused mild clinical manifestations in pwCF, suggesting that CFTR expression and function, or pharmacological treatments such as CFTR modulators might be involved and, in some way, influence the pathophysiology of COVID-19. This intriguing observation on the peculiarity of SARS-CoV-2 infection in pwCF prompts the investigation of the role of CFTR channel functions in COVID-19 pathophysiology.

### Cystic fibrosis and SARS-CoV-2

Viral respiratory infections are frequent in pwCF, being involved in more than 60% of pulmonary exacerbations. The level of susceptibility to viral infections along with the lung function deterioration usually present in pwCF led to the assumption of a possible greater risk of high incidence and severity of SARS-CoV-2 infection in this population [12, 13]. Some studies addressed the epidemiology, risk factors and severity assessment in pwCF. Table 1 summarises the main studies reporting the incidence of SARS-CoV-2 infection in pwCF. Overall, the incidence of infection in pwCF resulted lower compared to the general population in the same period.

**Table 1:** Incidence of SARS-CoV-2 infection in persons with Cystic Fibrosis (pwCF), January 2020 to April 2023

References	Study design	Number of patients	Period(s)	Main findings
Colombo C. et al. SARS-CoV-2 infection in cystic fibrosis: A multicentre prospective study with a control group, Italy, February-July 2020. PLoS ONE 2021, 16, e0251527. [14]	Multicenter prospective study conducted in Italy	6597	February–July 2020	pwCF had lower incidence of SARS-CoV-2 infection compared to the general population (2.4/1000 pwCF vs. 4.1/1000 inhabitants)
Naehrlich L. et al. Incidence of SARS-CoV-2 in people with cystic fibrosis in Europe between February and June 2020 J. Cyst. Fibros. 2021, 20, 566–577 [15]	Observational study within the European Cystic Fibrosis Society Patient Registry	130 PCR-confirmed cases	01 February 2020 and 30 June 2020, with data follow-up until 07 January 2021	The incidence of PCR-confirmed SARS-CoV-2 in pwCF (2.70 cases per 1000; 95% CI 2.25–3.20) was not statistically different to that of the general population (3.10/1000; 95% CI 3.10–3.11) Incidence was significantly higher in lung-transplanted pwCF (8.43/1000, 95% CI 5.35–12.62) versus non-lung transplanted pwCF (2.36/1000, 95% CI 1.94–2.86)
Mondejar-Lopez P. et al. Impact of SARS-CoV-2 infection in patients with cystic fibrosis in Spain: Incidence and results of the national CF-COVID19-Spain survey. Respir. Med. 2020,170, 106062 [16]	Retrospective observational study, based on the CF—confirmed COVID-19 Registry, Spain	2498	8 March–16 May 2020	Lower accumulated incidence in CF patients (32/10,000) than in the general population (49/10,000)
Padoan R. et al. First and second wave of SARS-CoV2 in Italian Cystic Fibrosis patients: Data from Italian Cystic Fibrosis Registry. Journal of Cystic Fibrosis 20 (2021) 372–373 [17]	Survey based-study involving the CF Centers located in Italy	5501	February-November 2020	First wave (February to August 2020) 22 CF patients were positive to SARS-CoV-2 virus i.e. 0.40% (0.23–0.57 CI95%) Second wave (September–November 2020) 65 CF patients i.e. 1.18% (0.90–1.47 CI95%)
Corvol H. et al. First Wave of COVID-19 in French Patients with Cystic Fibrosis J Clin Med. 2020 Nov 10;9(11):3624. [18]	Prospective observational study conducted in France	7500	1 March–30 June 2020	Lower incidence and overall risk reduction in pwCF with respect to the general population
Berardis S. et al. SARS-CoV-2 seroprevalence in a Belgian cohort of patients with cystic fibrosis. J. Cyst. Fibros. 2020, 19, 872–874. [19]	Prospective single center study conducted in Belgium	149	April 16, 2020–May 19, 2020	Lower seroprevalence in pwCF (2.7%) than in the Belgian population (4.3%) in the same period

Risk factors for severe COVID-19 in pwCF are reported in a recent systematic review [20]. Eight observational studies were included in the analysis, showing that the COVID-19 course was mild in most of pwCF [20]. Lung function deterioration, i.e. FEV1 < 40% predicted, was associated with higher hospitalization rate, whereas CF-related diabetes, pancreatic insufficiency and lung transplantation were identified as the main risk factors for severe outcomes. No significant effects on increased risk for severe outcome was associated to any genetic subgroup. The clinical impact of SARS-CoV-2 infection was milder in younger patients and the only treatment associated to a possible reduced risk was dornase alpha. Some recent evidence suggested a possible impact of COVID-19 on mental health of pwCF [21]. Noij et al. reported the psychosocial impact of COVID-19 highlighting an important risk of elevated depression and anxiety symptoms both in patients and caregivers. These results are consistent with a French study reporting an increased use of psychotic medications due to a high reported rate of symptoms related to anxiety (43.2%) and depression (51%) in the CF population in France during 2020 [22]. A single-center study report from Poland was not consistent with these results [23], as the authors did not find any significant effect of pandemic and lockdown measures on mental health of pwCF. Finally, the mid-term follow-up (6–12 months) studies on SARS-CoV-2 infection in pwCF indicated that the infection does not affect negatively respiratory outcomes [24, 25]. However, other evidence (Table 2) demonstrated that patients with pwFC had an increased risk of infection and severe COVID-19 symptoms, particularly in transplant patients, compared to the general population.In addition, in some studies subgroups of individuals with pwFC compared to the general population have significantly higher rates of hospitalisation. Hadi YB et al. showed that mortality, hospitalization, critical care need, mechanical ventilation, acute kidney injury and composite (combination of intubation and mortality) outcome at 30 days was higher in the pwCF, Jung A et al. reported that individuals with pwCF with forced expiratory volume in 1 s < 70% predicted, CFRD and those with lung transplantation are particularly at risk of more severe outcomes SARS-CoV-2 infectious disease.

**Table 2:** Incidence and outcome of SARS-CoV-2 infection in persons with Cystic Fibrosis (pwCF), November 2019 to January 2021

References	Study design	Number of patients	Period(s)	Main findings
Mathew HR, Choi MY, Parkins MD, Fritzler MJ. Systematic review: cystic fibrosis in the SARS-CoV-2/COVID-19 pandemic. BMC Pulm Med. 2021 May 20;21(1):173. https://doi.org/10.1186/s12890-021 [26]	Systematic review	339 individuals with CF who developed COVID-19	Between April 28 and December 10, 2020	within the CF population, the post-transplantation, may experience a more severe clinical course COVID-19
Hadi YB et al. Outcomes of SARS-CoV-2 infection in patients with cystic fibrosis: a multicenter retrospective research network study. Respir Med. 2021 Nov;188:106,606. https://doi.org/10.1016/j.rmed.2021.106606 [27]	A multicenter retrospective research network study	A total of 507,810 patients with COVID-19 were included (422 patients, 0.08% with CF; 507,388 patients, 99.92% without CF. Mean age at COVID-19 diagnosis in CF cohort was 46.6 ± 19.3 years, with female predominance (n = 225, 53.32%)	Between January 20, 2020, and January 09, 2021	In the crude, unmatched analysis, mortality, hospitalization, critical care need, mechanical ventilation, acute kidney injury and composite (combination of intubation and mortality) outcome at 30 days was higher in the pwCF
Jung A et al. Factors for severe outcomes following SARS-CoV-2 infection in people with cystic fibrosis in Europe. ERJ Open Res. 2021 Dec 27;7(4):00411–2021. https://doi.org/10.1183/23120541.00411-2021 [28]	Observational study	828 individuals with pwCF and SARS-CoV-2 infected	Between June 30, 2020 and December 31, 2020	SARS-CoV-2 infection yielded high morbidity and hospitalisation in pwCF. PwCF with forced expiratory volume in 1 s < 70% predicted, CFRD and those with lung transplants are at particular risk of more severe outcomes
McClenaghan E et al. The global impact of SARS-CoV-2 in 181 people with cystic fibrosis. J Cyst Fibros. 2020 Nov;19(6):868–871. https://doi.org/10.1016/j.jcf.2020.10.003 [29]	Observational study	181 people with CF	All the studies published from the beginning of the first case of COVID-19 (17 November 2019) to 13 June 2020	Infection with SARS-CoV-2 appears to exhibit a similar spectrum of outcomes to that seen in the general population, with 11 people admitted to intensive care (7 post-transplant), and 7 deaths (3 post-transplant). A more severe clinical course may be associated with older age, CF-related diabetes, lower lung function in the year prior to infection, and having received an organ transplant

## ACE-2 and CFTR channel, what is the correlation?

**Fig. 1:**
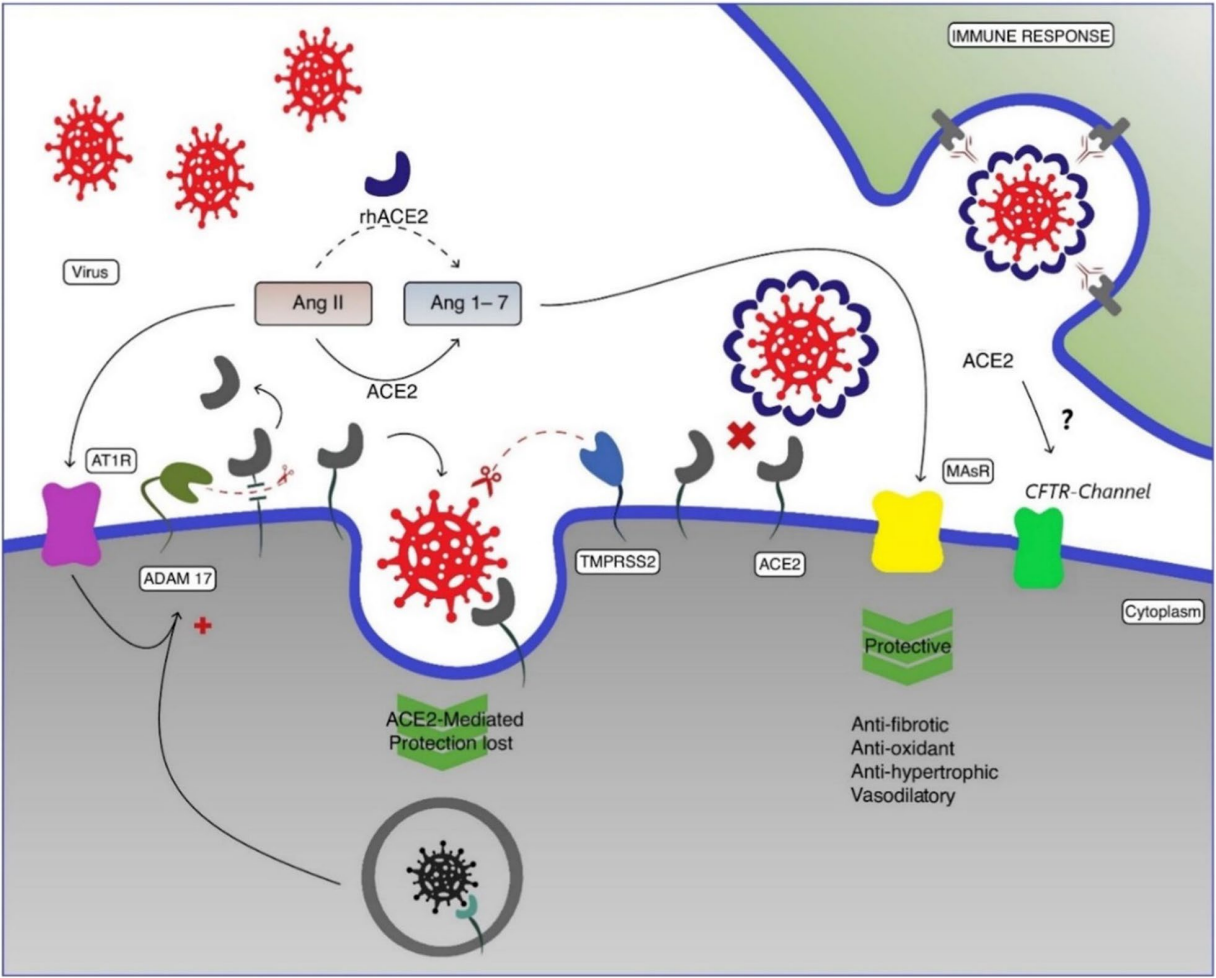
Severe acute respiratory syndrome coronavirus 2 (SARS-CoV-2) penetrates cells by binding between its peak protein (spike; S) to angiotensin-converting enzyme 2 (ACE-2). ACE-2 converts angiotensin (Ang)-II to Ang 1–7. The Ang1-7 has opposite biological actions to Ang-II (i.e., antifibrotic, antioxidant, antihypertrophic and vasodilatory effects) through stimulation of Mas receptor (MasR)

The SARS-CoV-2 virus uses ACE-2 for penetration into human cells by endocytosis and employs the cellular serine protease TMPRSS2 for priming the spike protein (S) [30, 31]. ACE-2 glycoprotein occurs in two forms: the first one is attached to the cell membrane (mACE-2) in several tissues of the human body, i.e. gut, kidney, heart, lungs; the second one is a soluble form (sACE-2) [32]. Both of these forms are part of the renin-angiotensin system (RAS), fulfilling important biological functions for the body's homeostasis. In the RAS system, the enzyme ACE-2 hydrolyses the carboxyl-terminal amino acid phenylalanine from angiotensin II (Ang-II) (Asp-Arg-Val-Tyr-Ile-His-Pro-Phe), converting it into angiotensin 1–7 (Ang 1–7) (H-Asp-Arg-Val-Tyr-Ile-His-Pro-OH), which stimulates Mas receptors (Mas-r). Consequently, Mas-r can induce vasodilator, anti-inflammatory, antifibrotic and antioxidant effects by antagonising the effects mediated by the AT-1r receptor stimulated by Ang II, such as vasoconstriction, fibrosis and thrombogenesis [33] (Fig. 1).

sACE-2 can cleave also numerous peptides, including [des-Arg9]-bradichinin, neurotensin, dynorphin A and ghrelin with important biological functions. ACE-2 and the RAS system, particularly the imbalance between Ang-II and Ang 1–7 can exert a central role in the pathogenesis of COVID-19 [34] complications. In view of its pivotal role as an entry receptor for SARS-CoV-2, it has been repeatedly hypothesised that intrinsic variation in ACE-2 expression may contribute to an individual genetic susceptibility to SARS-CoV-2 [35], several studies having reported that missense variants of ACE-2 may alter its affinity for the Spike protein, and consequently affect the endocellular penetration capacity of SARS-CoV-2 [36]. As described above, CF is caused by mutations in the CFTR gene, which encodes for proteins constituting a channel for chloride and bicarbonate that is widely expressed in respiratory epithelial cells, causing an alteration in mucociliary clearance and physiological homeostasis of the respiratory system. Because of their chronic pulmonary infections, which lead to respiratory failure, pwCF should be considered at high risk of developing severe symptoms of COVID-19. However, although pulmonary exacerbations caused by bacterial or viral infections are recurrent in CF, several epidemiological studies conducted on cohorts of patients with CF paradoxically demonstrated mild infections caused by SARS-CoV-2, with lower COVID-19-associated mortality rate than in the general population [32, 37]. The responsible factor could be the decreased or complete absence, depending on the type of CF class, of the CFTR channel protein that regulates other mediators involved in the pathophysiology of COVID-19 infection including ACE-2. Recent evidence showed that the CFTR channel protein may play an important role in regulating the expression and localisation of mACE-2, affecting cellular entry of SARS-CoV-2 [6]. In particular, mutations in the CFTR gene could cause a change in the pH of organelles in the protein secretion pathway, altering the glycosylation pattern of ACE-2 and/or TMPRSS-2 and consequently mitigate the effects of SARS-CoV-2 infection [3, 38]. To date, it is still not entirely clear whether the function of the CFTR channel protein, rather than its expression, can contribute to the replication defect of SARS-CoV-2 in respiratory cells of individuals with CF. It is likely that the correlation between CFTR and ACE-2 is not the only cause for the modulation of the viral infection and that other factors may play a protective role in pwCF infected by SARS-CoV-2.

## The role of CFTR modulators

Until 2012 the management of patients diagnosed with CF was mainly based on the use of drugs targeting the disease’ symptoms. With the discovery of the CFTR modulators, worldwide recognized as breakthrough therapies, a huge step forward was made in targeting the underlying cause. Indeed, CFTR modulators are small drugs that directly target the CFTR protein, coded by a long gene composed of 27 coding exons located on the long arm of chromosome 7. These drugs were developed to correct the malfunctioning protein made by the CFTR gene [39]. Disease-causing mutations can be classified into six categories that differ between each other for the type of defect. Specifically, the defect is in protein synthesis for Class I and V mutations, in protein trafficking for Class II, in channel gating for Class III, in channel conductance for Class IV and in plasma membrane protein stability for Class VI [40]. At this time, more than 2000 CFTR gene variants have been identified and individuals with CF may carry different CFTR mutations and, consequently, thousands of possible combinations of CF genotypes. For instance, many mutations can present features of more than one class [41], such as in the case of the well-studied F508del mutation that is classified as a class II mutation but exhibits feature of class III and IV defects as well. Therefore, the choice of the CFTR modulators should consider the overall genetic pattern [42]. Currently, four CFTR modulators have received the marketing approval: ivacaftor, elexacaftor, tezacaftor and lumacaftor. Ivacaftor was the first CFTR modulator to be approved in 2012. This drug is classified as a CFTR “potentiator” since it maintains for a longer amount of time the CFTR protein in an open state, by targeting CFTR mutations that impact channel gating [43]. A “potentiator” like ivacaftor is highly effective in the treatment of patients with CF carrying Class III and IV mutations. The drug, available as a single-agent product, is used in patients aged 4 months and above who have one of the following CFTR mutations: R117H, G551D, G1244E, G1349D, G178R, G551S, S1251N, S1255P, S549N and S549R. The remaining three CFTR modulator therapies are, instead, classified as “correctors” because they target the CFTR mutation F508del and improve CFTR protein conformation and subsequent processing and transfer to the cell surface [44]. The use of “correctors” is indicated in the treatment of patients with CF carrying Class II mutations. Ivacaftor is also available in a dual combination with tezacaftor that is indicated for patients aged 6 years and above who have inherited the F508del mutation from both parents or who have inherited the F508del mutation together with one of the following mutations: P67L, R117C, L206W, R352Q, A455E, D579G, 711 + 3A → G, S945L, S977F, R1070W, D1152H, 2789 + 5G → A, 3272 26A → G, or 3849 + 10kbC → T. Lumacaftor and tezacaftor were instead used in combination with ivacaftor in patients homozygous for F508del. Lastly, elexacaftor was combined to tezacaftor and ivacaftor to treat patients with at least one F508del variant (approximately 85% of CF cases). Data from clinical trials demonstrated the efficacy of ivacaftor, both as single-agent product and in dual and triple combinations, in improving respiratory function of patients with CF. For instance, the results of a phase III randomized controlled trial, which enrolled patients 12 years of age or older with at least one G551D-CFTR mutation, demonstrated that ivacaftor was associated with improvements in lung function starting from 2 weeks after the beginning of the treatment and that significant improvements were also observed in the risk of pulmonary exacerbations and patient-reported respiratory symptoms [45]. Similarly, the dual combination lumacaftor-ivacaftor significantly improved ppFEV1 and decreased pulmonary exacerbations in people with CF homozygous for F508del [46] Lastly, significant improvements in respiratory function were also observed during the triple combination therapy with elexacaftor-tezacaftor-ivacaftor (improvement of ppFEV1, respiratory symptom scores and decreased rates of pulmonary exacerbations) [47]. Regarding their safety profile, ivacaftor is commonly associated with the occurrence of respiratory adverse events (AEs), such as respiratory infection, hemoptysis, and acute respiratory failure, hepatic and gastrointestinal AEs, such as abdominal pain, nausea or vomiting, intestinal dysmotility, and gastroenteritis, headache and rash [48–51].Similarly, the dual combination lumacaftor/ivacaftor can be associated with the occurrence of respiratory AEs that included chest tightness, dyspnea, increased sputum, and declines in ppFEV1 [52–55].Lastly, as reported in the summary of product characteristics (SPC), compared to control groups, the triple combination therapy was more commonly associated to the occurrence of abdominal pain, diarrhea, rash and increases in liver enzymes and bilirubin [56]. Lastly, data from preclinical studies reported that CFTR expression/function is involved in the regulation of SARS-CoV-2 replication [57], while the results of a recent retrospective study reported that patients receiving the triple combination elexacaftor/ivacaftor/tezacaftor had a significantly decreased risk of developing acute respiratory failure after becoming infected with COVID-19 [3]. However, the role of CFTR, and consequently its pharmacological modulation, in the pathophysiology of COVID-19 disease is still not fully understood. Numerous evidences in the literature have shown that in severe stages of COVID-19 a dysregulated, multisystem inflammatory response induced by a cytokine cascade is responsible for the severe lesions of the disease [58]. Some evidence associates CFTR modulators with anti-inflammatory effects causing a significant reduction in sputum inflammatory markers in individuals with cystic fibrosis, including neutrophil elastase, IL-8 and IL-1beta [59]. Reducing inflammatory mediators might be helpful in avoiding the hyperinflammatory state that is generated in severe COVID-19 phases, however this might not be of benefit in the early stages of infection. Besides, there is good evidence that effective CFTR modulation can improve mucus clearance from the airways [60] representing a protective factor in preventing COVID-19 viral infections (Table 3).

**Table 3:** Potential CFTR Modulators positive effects in individuals with Cystic Fibrosis infected with SARS-CoV-2

CFTR modulators effect	References
Anti-inflammatory and significant reduction inflammatory mediators	Hisert KB et al. Restoring cystic fibrosis transmembrane conductance regulator function reduces airway bacteria and inflammation in people with cystic fibrosis and chronic lung infections. Am J Respir Crit CareMed. 2017; 195(12): 1617- 1628. https://doi.org/10.1164/rccm.201609-1954OC [61]
Improve airways mucus clearance	Altes TA et al. Use of hyperpolarized helium-3 MRI to assess response to ivacaftor treatment in patients with cystic fibrosis. J Cyst Fibros. 2017; 16(2): 267- 274. https://doi.org/10.1016/j.jcf.2016.12.004 [62]
Inhibition in vitro SARS-CoV-2 replication	Lotti V et al. CFTR Modulation Reduces SARS-CoV-2 Infection in Human Bronchial Epithelial Cells. Cells. 2022;11:1347. https://doi.org/10.3390/cells11081347 [63]
Significantly improved ppFEV1	Quittner AL et al. Development and validation of The Cystic Fibrosis Questionnaire in the United States: a health-related quality-of-life measure for cystic fibrosis. Chest 2005 Oct;128(4):2347–54. https://doi.org/10.1378/chest.128.4.2347 [46]
Significant improvements in respiratory function	Wainwright CE et al. Lumacaftor-ivacaftor in patients with cystic fibrosis homozygous for Phe508del CFTR. N. Engl. J. Med. 373:220–31. https://doi.org/10.1056/NEJMoa1409547 [47]

## Discussion

SARS-CoV-2 has shown high heterogeneity in spread and fatality rates between countries together with a significant variability in its clinical presentation, indicating that host genetic, clinical, demographic, geographic and behavioural determinants and interactions may influence its pathogenicity. Since the beginning of the pandemic, some scientific evidence reported correlation between pwCF and lower incidence of SARS-CoV-2 infection compared to the general population. Besides, CF appeared a protective factor in terms of COVID-19-associated mortality and severity. Conversely, subjects carrying single pathogenic variants of the CFTR gene, i.e. CF carriers, more likely underwent severe COVID-19 with high risk of 14-day mortality. These evidences link inversely the degree of expression of the CFTR channel and COVID-19 severity, pointing to a molecular biological plausibility corroborating the role of CFTR in the pathogenesis of SARS-CoV-2 infection. Moreover, ACE-2 mRNA expression was significantly reduced in both primary human bronchial epithelial cells and nasal epithelial cells isolated from patients affected by CF, leading to impaired SARS-CoV-2 cell entry and replication [64]. Similarly, Lotti et al. detected no major difference in ACE-2 expression before infection between wild-type and CFTR-modified cells in vitro, while higher ACE-2 expression in wild-type compared to CFTR-modified cells after infection allowed viral replication in the former but not in the latter [3]. Also, pharmacological inhibition of CFTR activity limits SARS-CoV-2 infection, i.e. by means of CFTR modulators, while mACE-2 expression in the nasal epithelium of paediatric patients has been shown lower than in adults, explaining their mild/asymptomatic clinical evolution of SARS-CoV-2 infection. Thus, host genetic and phenotypic factors were shown involved in determining COVID-19 presentation and progression, including CFTR channel and ACE-2 expression [65]. Other angiotensins involved in the processing of Ang-II into Ang1,7 could influence the interactions between Spike proteins (both from SARS-CoV-2 and vaccine-associated) and ACE-2 receptors [23–26]. Understanding the relationships between the different mechanisms of Ang-II cleavage and accumulation offers the opportunity to delineate a unique pathophysiological mechanism that explains the risk of progression to severe forms of COVID-19 and potential AEs following vaccination. However, evidence showed that CFTR channel inhibition in vitro, mimicking the conditions of CF, can influence SARS-CoV-2 replication [3]. On the other hand, in individuals with CF a modulation of CFTR with increased opening frequency, and thus a restoration of the channel function with modulating agents, may result in an improvement of mucociliary clearance, lung microbiota, acting as defence mechanisms against severe COVID-19. To date, the protective factor against COVID-19 in patients with CF, and the role and correlation of CFTR and ACE-2, and of CFTR modulating agents, is not fully elucidated.

## Conclusions

Current evidence suggests that pwCF are likely to have a lower incidence and milder course of COVID-19 than the general population. The mechanisms responsible have yet to be fully defined. An important role is played by the level of expression of CFTR, its linkage with ACE-2, and the indirect effects of therapies in pwCF. The use of pharmacological treatments, such as CFTR modulators, should be evaluated in pwCF in the light of the risk of severe disease due to SARS-CoV-2. However, caution is needed in the interpretation of available evidence that needs to be supported by further investigations.

## Disclosure

The authors declare they have used neither AI nor AI-assisted technologies in this work.

## Data Availability

Full availability of data and materials.
